# mSphere of Influence: Combining Host and Pathogen Genetics To Disrupt Chronic Infections

**DOI:** 10.1128/mSphere.00106-20

**Published:** 2020-02-26

**Authors:** Andrew J. Olive

**Affiliations:** a Michigan State University, East Lansing, Michigan, USA

**Keywords:** *Mycobacterium*, *Mycobacterium tuberculosis*, genetics, immune evasion, persistent infections, transposon sequencing

## Abstract

Andrew J. Olive works in the field of host responses to chronic infections. In this mSphere of Influence article, he reflects on how “Tryptophan biosynthesis protects mycobacteria from CD4 T-cell-mediated killing” (Y. J. Zhang, M. C. Reddy, T. R. Ioerger, A. C. Rothchild, et al., Cell 155:1296–1308, 2013, https://doi.org/10.1016/j.cell.2013.10.045) impacted his own work using genetic approaches to dissect the interface between host and pathogen.

## COMMENTARY

During infection, Mycobacterium tuberculosis can evade and withstand the stresses imposed by the host immune response (1, 2). Understanding the precise mechanisms used by M. tuberculosis to survive an ongoing immune response in the host remains key to effectively developing new treatment options. In 2013, Zhang et al. published “Tryptophan biosynthesis protects mycobacteria from CD4 T-cell-mediated killing” (3). The central finding from that paper is that M. tuberculosis must synthesize its own tryptophan in mammalian hosts with functional CD4^+^ T cells to cause disease. While these findings were important on their own, the greater impact of this paper stems from the global approaches that provided a path to understanding distinct mechanisms that control tuberculosis (TB) disease at the host-pathogen interface.

To identify the M. tuberculosis genes that are required only in the presence of CD4^+^ T cell-mediated immunity, Zhang et al. used a forward genetic approach. A pooled transposon library was used in M. tuberculosis to infect wild-type C57bl6 mice and mice deficient in CD4^+^ T cell responses (major histocompatibility complex class II deficiency [MHCII^−/−^]). M. tuberculosis genes that were differentially required in these two strains were identified by transposon insertion sequencing (Tn-seq). By comparing the genes required in mice with functional CD4^+^ T cells with results of *in vitro* studies examining predicted stress conditions, the authors found that the M. tuberculosis tryptophan biosynthesis pathway is conditionally essential in mice with functional CD4^+^ T cells. Given that many intracellular pathogens are tryptophan auxotrophs, it was well known that CD4^+^ T cell-derived gamma interferon (IFN-γ) restricts tryptophan availability by inducing the tryptophan-catabolizing enzyme indolamine 2,3-dioxagenase (IDO) (4, 5). Using a combination of *in vivo* and *ex vivo* experiments, the authors carefully showed that an M. tuberculosis mutant with a mutation in the tryptophan synthesis pathway (ΔTrpE) was unable to survive IFN-γ-induced IDO expression in a CD4^+^ T cell-dependent manner in both mouse and human cells. This effect could be reversed by increasing the exogenous tryptophan concentration, showing that M. tuberculosis must synthesize tryptophan *de novo* in hosts with a functional immune system. To test whether inhibition of M. tuberculosis tryptophan synthesis is a viable therapeutic strategy, the authors identified small molecules inhibitors. They found the anthranilate analog 6-FABA synergistically killed M. tuberculosis with IFN-γ/tumor necrosis factor alpha (TNF-α) stimulation and reduced M. tuberculosis growth *in vivo*. Thus, by defining mechanisms that allow M. tuberculosis survival during active host immune responses, the authors provided a proof of the principle that targeting immune evasion genes is a realistic therapeutic possibility.

The underlying concepts in the study by Zhang et al. reminded me of a review by Persson and Vance (6) that I read early in my graduate career. In that review, the authors discussed the advantages of combining bacterial and host genetics, or “genetics-squared,” to examine host-pathogen interactions. I was struck by the argument that genetic approaches can be used to tease apart tightly linked interactions between the pathogen and the host, and I was inspired to use this approach in my own research. At the time, I was studying Chlamydia trachomatis with a limited genetic toolbox that prevented me from directly using genetic-squared approaches; constraints in genetic editing in *Chlamydia* meant that projects requiring specific mutants became dead ends. For my postdoctoral work, I wanted a genetically tractable pathogen that would allow me to combine both host and bacterial genetic approaches. As I was completing my graduate training in 2013, Zhang et al. published their study, and it profoundly influenced the questions that I would pursue as a postdoctoral fellow and beyond. When I read the paper by Zhang et al., I saw it as a guide for dissecting the genomes of pathogens in immune-defined hosts. Using a library of M. tuberculosis mutants as reporters of the environment that bacteria encounter under distinct immune stress conditions or in immune-deficient animals was a powerful approach. While several studies had determined these types of host-pathogen genetic interactions on a candidate basis, the speed and scale at which Zhang et al. completed these studies improved the ability to identify important genes in an unbiased manner in this field (7–10). Additionally, the premise and conclusion of their study were simple and yet profound: the mycobacterial genes that drive immune evasion could be therapeutic targets that, when blocked, could improve M. tuberculosis control. That study inspired me to transition to M. tuberculosis research for my postdoctoral training. It became clear to me that there was a lack of understanding of how host protective pathways mechanistically function to control M. tuberculosis growth and survival (1). I performed bacterial forward genetic screens under a broad range of immune conditions to begin to dissect how M. tuberculosis withstands so many distinct host immune mechanisms. For example, using genome-wide bacterial genetic approaches, we examined how bacterial requirements change in distinct immune knockout animals, such as Nos2^−/−^ animals, that are unable to make nitric oxide (11). We found that hyperinflammation in Nos2 animals creates a growth-permissive environment, relieving the need for many stress response and nutrient acquisition genes. Several other groups have published similar approaches in different organisms, highlighting how generalizable these host-pathogen genetic interaction studies are and the impact that Zhang et al. had on the field (12–14).

Over the coming years, it is likely that we will continue to see many studies that combine host and pathogen genetics to understand the mechanisms of infectious disease. Not only will forward genetic bacterial screens be performed in defined knockout hosts, but there will likely be an increase in forward host genetic screens using defined bacterial mutants. This strategy was recently published in a tour de force study by Xu et al. that showed the power of host genetic CRISPR approaches to examine the host pathways that drive distinct stress conditions during infection (15). To date, most studies have used only a genome-wide bacterial library and a defined set of host mutants. However, as dual host and pathogen transcriptome sequencing (RNA-seq) is now widespread, it is not difficult to imagine dual host and pathogen genome-wide screening as a real possibility in the near future (16, 17). This technological advance would further the impact of the study by Zhang et al. and would take the genetics-squared approach to the next level by allowing a deeper understanding of how pathogens manipulate immunocompetent hosts and the development of new strategies to treat these infections.
